# Supplementary material for: Deletion of wfs1 Impairs Oligodendrocyte Precursor Cells Dorsal Distribution and Myelination Through the wfs1-hmgcs1 Axis in Zebrafish
Source: Biology (Basel). 2026 Aug 16;15(16):1402. doi: 10.3390/biology15161402 (PMC13510151; doi:10.3390/biology15161402)
Supplement: Supplementary file 1 [file biology-15-01402-s001.zip › File S1-Transcriptome sequencing data and differentially expressed genes (DEGs) in wfs1-- zebrafish at 5 dpf/1.GO/wfs1KOvsWT/DOWN/wfs1KOvsWT.down.GOmf_DAG.pdf]

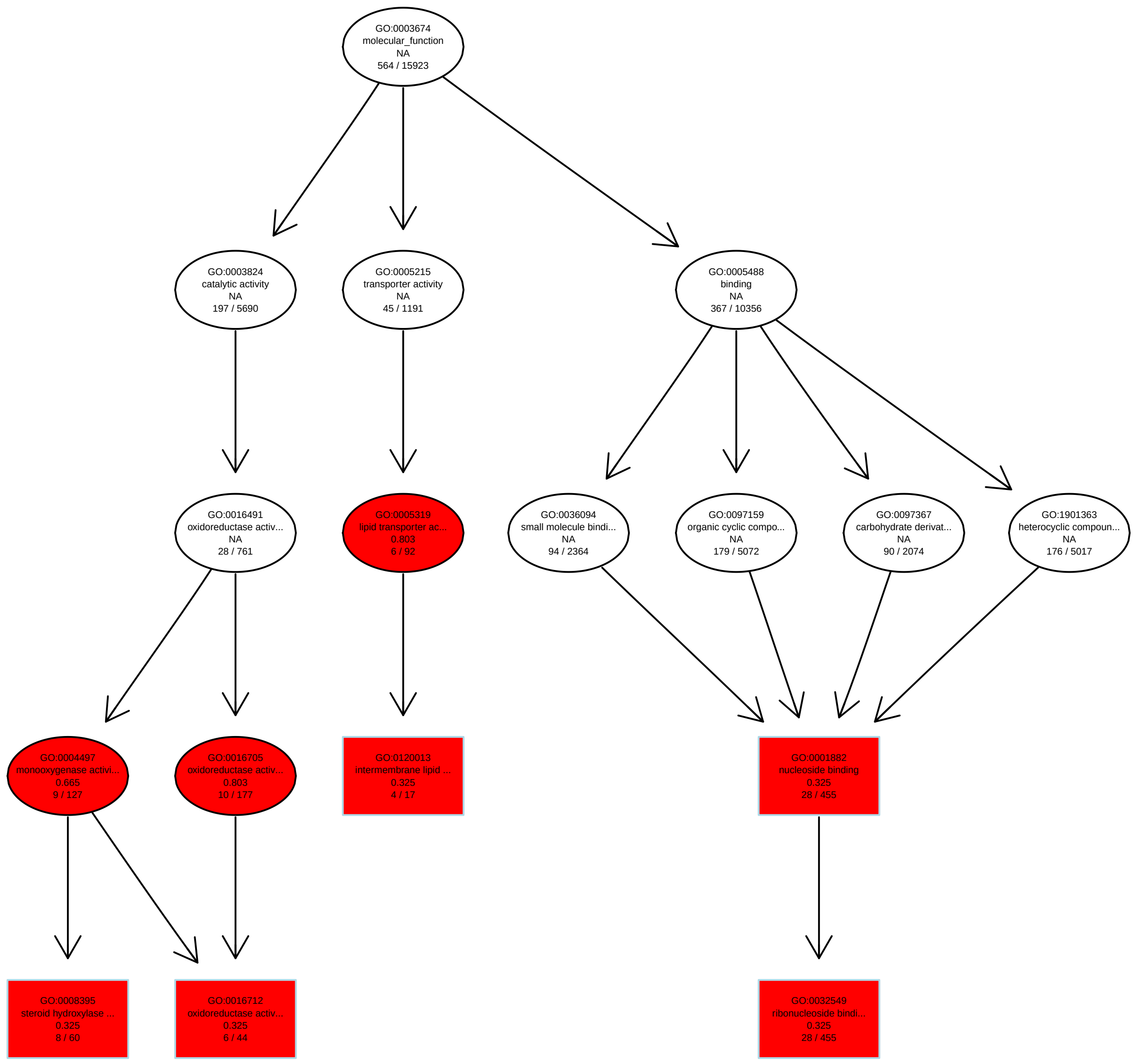
GO:0003674
molecular_function
NA
564 / 15923
GO:0003824
catalytic activity
NA
197 / 5690
GO:0005215
transporter activity
NA
45 / 1191
GO:0005488
binding
NA
367 / 10356
GO:0016491
oxidoreductase activ...
NA
28 / 761
GO:0005319
lipid transporter ac...
0.803
6 / 92
GO:0036094
small molecule bindi...
NA
94 / 2364
GO:0097159
organic cyclic compo...
NA
179 / 5072
GO:0097367
carbohydrate derivat...
NA
90 / 2074
GO:1901363
heterocyclic compoun...
NA
176 / 5017
GO:0004497
monooxygenase activi...
0.665
9 / 127
GO:0016705
oxidoreductase activ...
0.803
10 / 177
GO:0120013
intermembrane lipid ...
0.325
4 / 17
GO:0001882
nucleoside binding
0.325
28 / 455
GO:0008395
steroid hydroxylase ...
0.325
8 / 60
GO:0016712
oxidoreductase activ...
0.325
6 / 44
GO:0032549
ribonucleoside bindi...
0.325
28 / 455
